# Soil salinity regulates spatial-temporal heterogeneity of seed germination and seedbank persistence of an annual diaspore-trimorphic halophyte in northern China

**DOI:** 10.1186/s12870-024-05307-x

**Published:** 2024-06-26

**Authors:** Zhaoren Wang, Jerry M Baskin, Carol C Baskin, Guofang Liu, Xuehua Ye, Xuejun Yang, Zhenying Huang

**Affiliations:** 1 State Key Laboratory of Vegetation and Environmental Change, Institute of Botany, the Chinese Academy of Sciences, Beijing, 100093 China; 2 Department of Biology, University of Kentucky, Lexington, KY 40506 USA; 3 Department of Plant and Soil Sciences, University of Kentucky, Lexington, KY 40546 USA; 4 Institute of Urban Environment, Chinese Academy of Sciences, Xiamen, 361021 China

**Keywords:** *Atriplex Centralasiatica*, Environmental heterogeneity, Seed burial depth, Seed dormancy/Germination, Seed heteromorphism, Soil salinity, Soil seedbank

## Abstract

**Background and aims:**

Seed heteromorphism is a plant strategy that an individual plant produces two or more distinct types of diaspores, which have diverse morphology, dispersal ability, ecological functions and different effects on plant life history traits. The aim of this study was to test the effects of seasonal soil salinity and burial depth on the dynamics of dormancy/germination and persistence/depletion of buried trimorphic diaspores of a desert annual halophyte *Atriplex centralasiatica*.

**Methods:**

We investigated the effects of salinity and seasonal fluctuations of temperature on germination, recovery of germination and mortality of types A, B, C diaspores of *A. centralasiatica* in the laboratory and buried diaspores in situ at four soil salinities and three depths. Diaspores were collected monthly from the seedbank from December 2016 to November 2018, and the number of viable diaspores remaining (not depleted) and their germinability were determined.

**Results:**

Non-dormant type A diaspores were depleted in the low salinity “window” in the first year. Dormant diaspore types B and C germinated to high percentages at 0.3 and 0.1 mol L^-1^ soil salinity, respectively. High salinity and shallow burial delayed depletion of diaspore types B and C. High salinity delayed depletion time of the three diaspore types and delayed dormancy release of types B and C diaspores from autumn to spring. Soil salinity modified the response of diaspores in the seedbank by delaying seed dormancy release in autum and winter and by providing a low-salt concentration window for germination of non-dormant diaspores in spring and early summer.

**Conclusions:**

Buried trimorphic diaspores of annual desert halophyte *A. centralasiatica* exhibited diverse dormancy/germination behavior in respond to seasonal soil salinity fluctuation. Prolonging persistence of the seedbank and delaying depletion of diaspores under salt stress in situ primarily is due to inhibition of dormancy-break. The differences in dormancy/germination and seed persistence in the soil seedbank may be a bet-hadging strategy adapted to stressful temporal and spatial heterogeneity, and allows *A. centralasiatica* to persist in the unpredictable cold desert enevironment.

**Supplementary Information:**

The online version contains supplementary material available at 10.1186/s12870-024-05307-x.

## Introduction

Halophytes (salt-tolerant plants) thrive in saline conditions that kill 99% of glycophytes (salt-intolerant plants), and they have evolved mechanisms to cope with high salt concentration in the soil [1]. Tolerance of halophytes to high salinities such as those that occur in seasonally-dry habitats involves a delay of seed germination until salinity is decreased by precipitation, at which time seeds can germinate and seedling become established [2–4]. That is, germination is prevented by high salinity, but when the salinity is decreased by input of fresh water seeds can recover from exposure to high salinity and germinate [5].

Soil seedbanks, which include all viable seeds on or in the soil, represent a critical but hidden stock for the long-term survival of plant populations and plant community dynamics [6–8]. Dormancy is defined as the inability of viable seed to germinate under favorable conditions, such as water, temperature, light and gases, and can be divided into physiological dormancy (PD), morphological dormancy (MD), morphophysiological dormancy (MPD), physical dormancy (PY), and physical plus physiological dormancy (PY + PD) [7]. Seed with nondeep physiological dormancy (PD), may exhibit an annual dormancy (D)/nondormancy (ND) cycle during burial in soil in response to seasonal changes in environmental conditions in the habitat, dormancy cycles are often associated with plants that inhabit environments with strong seasonal fluctuations in temperature, rainfall, or other environmental conditions [7, 8]. . Dormancy cycling helps to control timing of germination to occur when conditions in the habitat are optimal for seedling establishment and survival [7, 9]. Many laboratory studies have shown that salinity can either inhibit seed germination or induce ND seeds into D [7, 10, 11]. However, the degree of soil salinity also cycles annually in habitats, but how such cycles affect seed dormancy cycle relates in saline habitats is still not clear.

Seed heteromorphism, the production on an individual plant of two or more types of seeds that differ in morphology and ecology, is common in halophyte genera, such as *Arthrocnemum*, *Atriplex*, *Chenopodium*, *Salsola*, and *Suaeda* [1, 12]. Production of heteromorphic seeds with different germination and dormancy responses is an adaptive strategy of plant species in saline areas, which confers a selective advantage for plants in extreme and fluctuating environments [7, 13, 14]. Seed heteromorphism may function as a bet-hedging strategy by spreading germination risks in time and space in unpredictable habitats [15–17]. Despite many studies on adaptive strategies of heteromorphic seeds in different salinity and burial conditions [10, 18–22], nothing is known about how soil salinity and burial conditions in the field affect germination and persistence of seeds in the seedbank.

*Atriplex centralasiatica* is a C_4_ summer annual halophyte widely distributed in arid and semi-arid saline cold desert habitats in northern China [23, 24]. Soil salinity in habitats of *A. centralasiatica* fluctuates seasonally in response to variations in precipitation and drought [10, 11]. *A. centralasiatica* is a halophytic plant species that is well-adapted to growing in salt desert environments. *A. centralasiatica* is important source of nutrition as forage for livestock in these areas [23]. According to Zhang et al. [14] *A. centralasiatica* produces two kinds of diaspores, but Wang et al. [11, 25] found that three types of diaspores are produced by this species: (1) type A, flat, relatively small with yellow inner utricle, fast germinating (non-dormant), high salt tolerance, depleted from the (transient) seedbank 7 months after shedding; (2) type B, flat, intermediate size with black inner utricle, nondeep PD, salt tolerant, depleted from the (short-lived persistent) seedbank in 20 months after shedding; and (3) type C, relatively large and globular with black inner utricle, deeper degree of nondeep PD than diaspore type B, low salt tolerance, persistent soil seedbank. Each type of diaspore is enclosed by a bracteole when it is dispersed [11]. Populations of *A. centralasiatica* only inhabit in environments with cold and dry winter [23]. The diaspore collection area has a typical continental, semi-arid climate with mean monthly temperatures that range from − 8.2 °C in January to 23.0 °C in July; mean annual precipitation of 254.3 mm with most of the precipitation falling in later spring and summer (see Figure S1).

We hypothesized that the seasonal dynamics of soil salinity play a crucial role in regulating seed dormancy/ germination and long-term viability of the soil seedbank of *A. centralasiatica*, thereby influencing these life-history traits. We ask the following questions: (1) How do the three diaspores morphs respond to the seasonal fluctuations in soil salinity in the field? (2) What is the influence of soil salinity and burial depth on dormancy/germination and longevity of diaspores in the soil seedbank? To test this hypothesis, we determined the effects of soil salinity, burial depth and their interaction on the three types of diaspores of *A. centralasiatica*. We predicted that the trimorphic diaspores respond differently to soil salinity and burial depth, which may further affect the time of seedling establishments.

## Materials and methods

### Study area and diaspore collection

Freshly mature diaspores of *A. centralasiatica* were collected from natural populations growing on the edge of salt lakes on the Ordos Plateau of Inner Mongolia, northern China (38° 15′ 14′′ N, 107°es 28′ 52′′ E, 1314 m a.s.l.), on 20 September 2016. Persistent bracteoles enclose each inner seed firmly, especially for types B and C diaspores. The inner seed was still shiny when removed from the diaspores after the 2–year experiment. Diaspores were air-dried under laboratory conditions for 1 week and then stored at -20 °C for subsequent experiments. The plant samples were identified by Zhenying Huang, a botanical professor at Institute of Botany, the Chinese Academy of Science. Note that this species is widely distributed in the salt deserts and coastal zones of northern China. Therefore, collection permission is not required, and a voucher specimen of the plant materials were deposited in Ordos Sandland Ecological Research Station of the Chinese Academy of Sciences. The experimental research conducted on plants in this study adhered to institutional, national, and international guidelines.

### Number of diaspores at different soil depths in the field

Ten soil samples 10 cm long x 10 cm wide x 10 cm deep were collected in November 2016 (most freshly matured diaspores dispersed), March 2016 (beginning of germination season) and mid-August 2017 (end of growing season but before dispersal of freshly-matured diaspores), in the natural habitat of *A. centralasiatica*. The number of diaspores in the 0–2, 2–5 and 5–10 cm layers of soil in each sample was counted.

### Effect of salinity on germination and recovery of germination

The effect of salt on germination of freshly mature types A, B and C diaspores and diaspore types B and C stored dry (~ 30% relative humidity, and ~ 20 °C) for 6 months and then given 8 weeks of cold stratification (moist sand with 12% water content at 4 °C) to break dormancy) were tested. Germination was tested at 5/15°C (12/12 h) in 12 h light (approx. 100 µmol m^− 2^ s^− 1^, cool white fluorescent light) and 12 h darkness, which approximates the temperature regime in April when seeds germinate in the habitat and the optimal temperature regime for seed germination, at 0 (distilled water, CK), 0.05, 0.1, 0.2, 0.4, 0.8 and 1.5 mol L^− 1^ NaCl. After 30 days of NaCl treatments, non-germinated diaspores were rinsed three times with distilled water and then incubated in light at 5/15°C (the best conditions for germination) for 30 days in Petri dishes on filter paper moistened with 2.5 mL distilled water. Recovery percentages were calculated as [(a − b)/c] × 100, where a is number of diaspores that germinated in salt solution plus the quiescent diaspores that germinated after they were incubated in NaCl solutions for 30 days and then transferred to distilled water for 30 d; b number of diaspores germinated in salt solution; and c total number of diaspores tested [11]. The viability of non-germinated diaspores was tested by the tetrazolium method. We separated embryos from diaspores and soaked them in TTC solution for two hours, and inner seeds with uniform and intense colour are determined as viable ones.

### Burial of heteromorphic diaspores at different soil depths and salinities

**Fig. 1:**
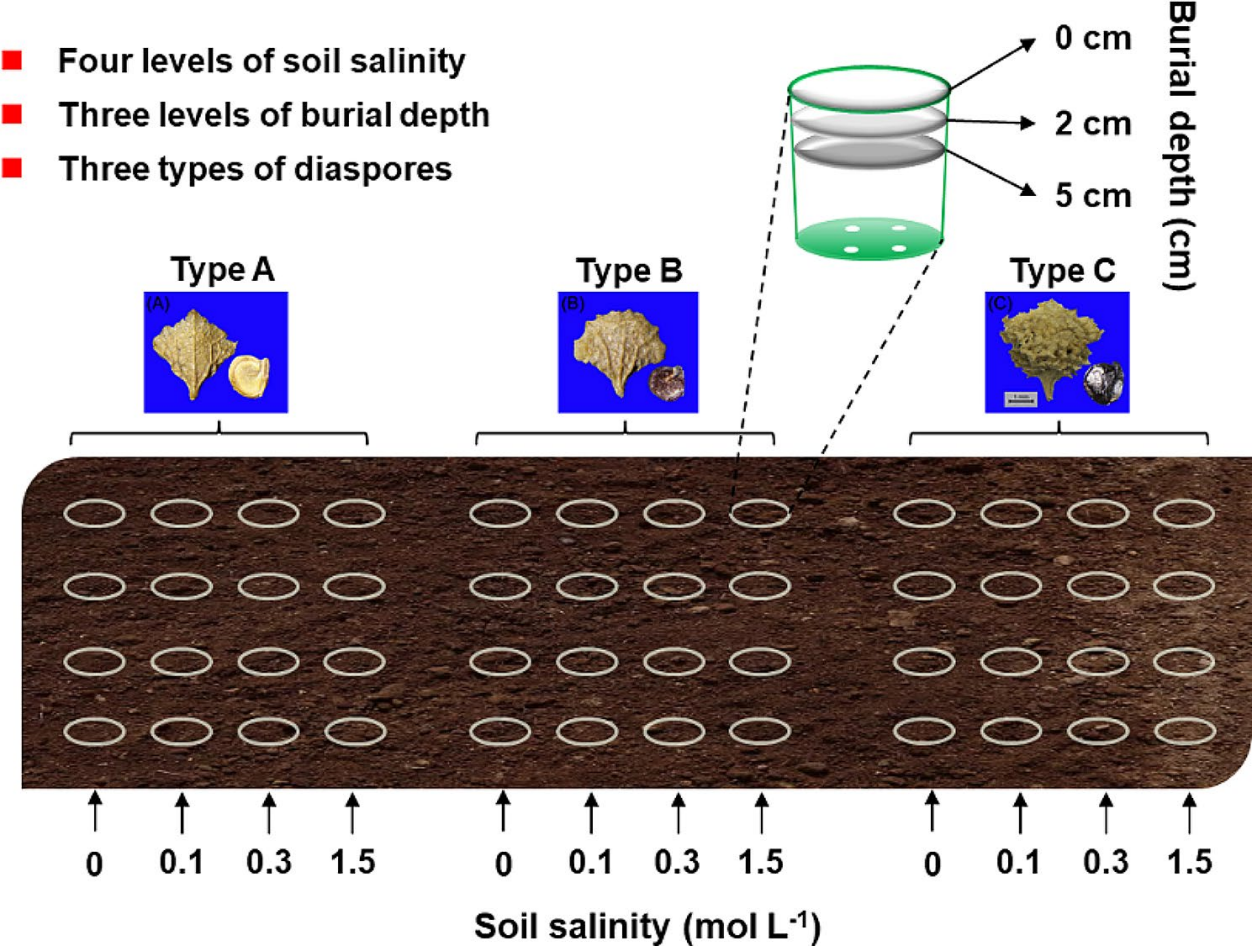
Experimental design for effects of soil salinity and burial depth on germination responses and persistance in the seedbank of the three diaspore types of *Atriplex centralasiatica*. L

The experiments were carried out near the Ordos Sandland Ecological Research Station of the Chinese Academy of Sciences, where climatic conditions are the same as those at diaspore collecting sites. Each of the three types of 800 freshly mature diaspores of *A. centralasiatica* was placed in nylon-mesh bags and buried in sand at 0, 2 and 5 cm in each plastic pots (diameter, 55 cm; height, 70 cm) for 24 months, 20 December 2016 to November 2018. Four holes 5 cm in diameter were made in the bottom of each pot to allow for drainage of excess water from the pots. NaCl is the main salt in soil of the natural habitats of the experimental sites [10]. Soil salinity (NaCl) applied to the pots was 0, 0.1, 0.3 and 1.5 mol L^-1^, which represent control, mean level in the field, high level in the field and extreme condition that diaspores dispersed into the dry bed of the salt lake would encounter, respectively. Forth-eight pots were filled with clean sand with electroconductibility less than 0.01 mol L^-1^ NaCl equivalent and then mixed with the four concentrations of NaCl solution. Thus, 48 pots with 3 types of diaspores × 3 burial depths (in each pot) × 4 levels of salinity × 4 replicates were used (Fig. 1). All pots were buried on level ground, with the top of the pot even with the surface of the sand.

Soil temperature, moisture and salinity in each pot with different burial depths and salinities during the study were recorded every 2 h by data collectors (Em50 METER Co. Ltd, USA). Soil moisture and salinity were not measured for November, December, January and February, since the soil was frozen. To replenish the salt that flowed out the holes in the pots, NaCl were added to the set level each October according to the salt concentration measured by the EM50. Diaspores in the nylon-mesh bags were buried in December 2016, since most diaspores of *A. centralasiatica* are dispersed after the snowfall in December [11].

### Dynamics of potential germination and depletion of heteromorphic diaspores

Diaspores of the three morphs in nylon-mesh bags in each pot were exhumed on the 20th day of each month for 24 months, from December 2016 to November 2018. The remaining diaspores were counted after removing the germinated ones (radicle emerged or only empty bracteoles left in each bag). Germination was tested on two filter papers moistened with 3 ml distilled water at 5/15, 10/20, 15/25 and 20/30°C (12/12 h) in 12 h light (approx. 100 µmol m^− 2^ s^− 1^, cool white fluorescent light) and 12 h darkness. The alternating temperature regimes represent the approximate mean daily minimum and maximum air temperatures for each month during the growing season at the seed collecting site: 5/15, April and October; 10/20, May and September; 15/25, June and August; and 20/30°C, July (World Climate, www.worldclimate.com). Four replicates of 25 diaspores (or one-fourth of remaining diaspores in the bags if the number was not enough) each was used in each test. Final germination was determined after 30 d. Emergence of the radicle was the criterion for germination. After 30 d germination tests, non-germinated diaspores were examined to determine whether the embryo was firm, indicating that they were viable, or soft and with yellow liquid exuded, indicating that they were nonviable. Tetrazolium tests confirmed that the firm embryos were viable and the soft ones non-viable [26]. Therefore, data on the number of remaining diaspores, dynamics of dormancy/germination and number of diaspore deaths were collected. Only viable diaspores were used in calculating germination percentages.

### Statistical analyses

All data analyses were done with R software (v3.3.2, R Core Team 2016). The response variables were expressed as mean ± SE or mean + SE (non-transformed data appear in all figures). A general linear model (GLM) with family = binomial (‘logit’) model [27] was used to test whether different experimental treatments, including soil salinity, temperature, time and three types of diaspores as variables, diaspore germination as response variables, significances on germination followed by multiple contrasts with Tukey’s HSD tests using the ‘glht’ function of the ‘multcomp’ package. Level of significance was set at *P* < 0.05.

## Results

### Number of diaspores at different soil depths in the field

**Fig. 2:**
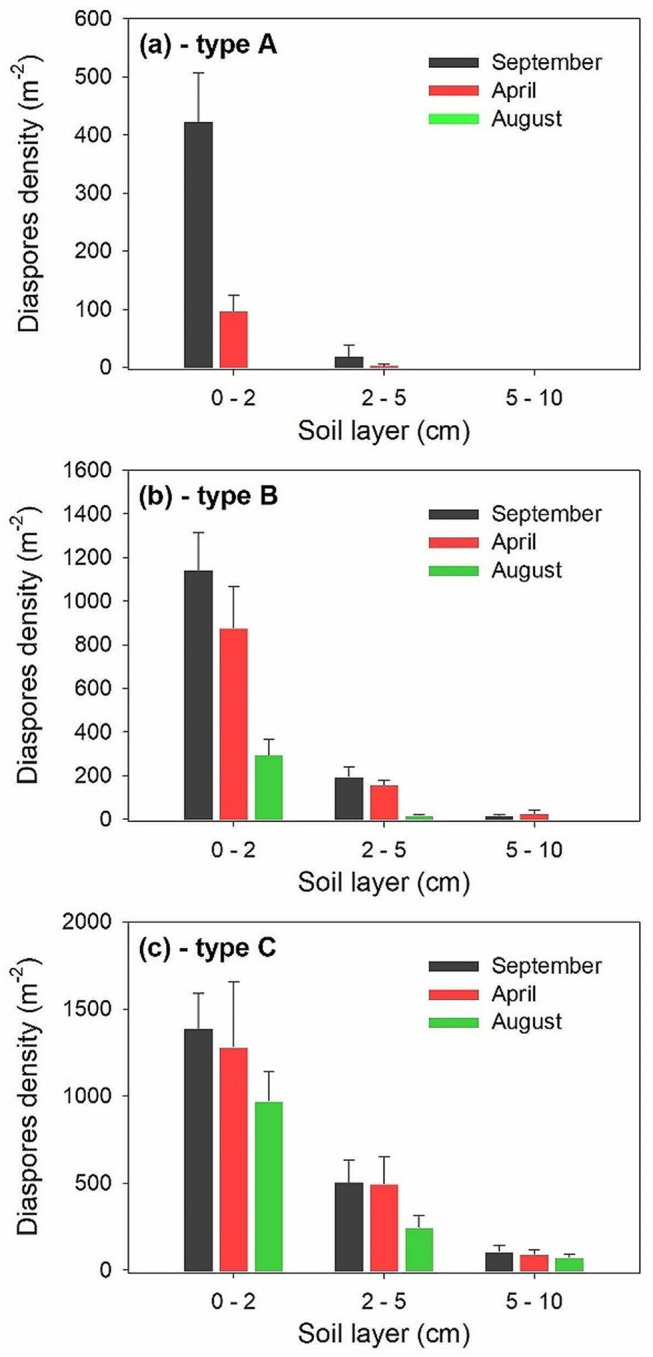
Sizes (mean ± SE) of in situ soil seedbank of the trimorphic diaspores of *Atriplex centralasiatica* on 30 September, 1 April and 20 August, which are the end of the life cycle, beginning of seedling establishment season and end of the growing season but prior to time for diaspore dispersal, respectively

In the habitat of *A. centralasiatica*, most diaspores of types A, B and C were distributed in the 0–2 cm soil layer of in September, April and August, especially for types A and B diaspores (Fig. 2). For type A diaspores, only 22.76% remained in the soil seedbank in early April, the beginning of the germination season, and all of them had been depleted at the end of growing season in August (Fig. 2a). Depletion of most types B (Fig. 2b) and C (Fig. 2c) diaspores occurred mainly in the germination season, especially at the depths of 0–2 and 2–5 cm. However, compared with September, over 93.23% and 64.56% type C diaspores remained in the soil seedbank in early April and mid-August, respectively.

### Effect of salinity on germination and recovery of germination

**Fig. 3:**
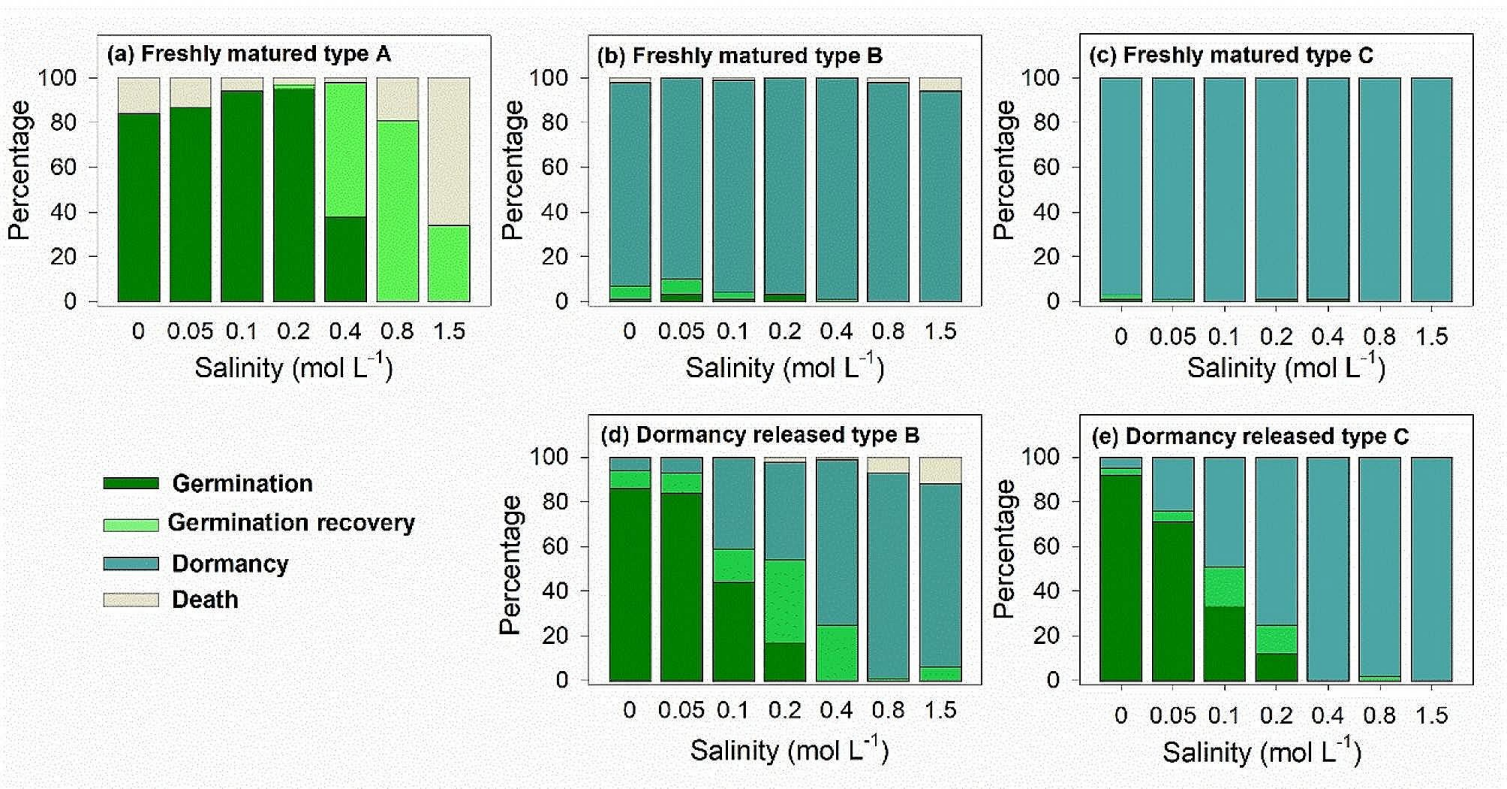
Mean percentage germination, germination recovery, dormancy and nonviable freshly matured A (**a**), B (**b**) and C (**d**) diaspores and dormancy-released B (**c**) and C (**e**) diaspores of *Atriplex centralasiatica* incubated in various concentrations of NaCl at 5/15°C. Germination recovery is quiescent diaspores that did not germinate after 30 days of incubation in the NaCl solutions but did germinate when moved from salt solutions to distilled water

Freshly matured type A diaspores germinated to > 80% when incubated in NaCl solutions with a concentration of less than 0.2 mol L^-1^ (Fig. 3a). At 0.4 mol L^-1^, 38% of type A diaspores germinated, and the other 59% did so after transferring them to distilled water for 1 month of incubation. No type A diaspores germinated at 0.8–1.5 mol L^-1^, but they germinated to 81% and 34%, respectively, after being moved to distilled water for 1 month. All non-germinated diaspores were nonviable. Less than 10% of freshly matured types B (Fig. 3b) and C (Fig. 3c) diaspores germinated at each concentration of incubation solution. Germination of dormancy-released diaspore types B (Fig. 3d) and C (Fig. 3e) decreased sharply with salinity between 0 and 0.40 mol L^-1^ NaCl, especially those of type C. Type B diaspores had higher germination recovery than those of type C, which had low germination recovery. Most diaspore types B and C were viable after the recovery tests.

### Soil temperature, moisture and salinity

Soil temperature, moisture and salinity fluctuated with time (Fig. S1). Soil temperature increased from spring to early summer and decreased from summer to winter (Fig. S1). Soil salinity increased from late summer to winter and spring due to the increased evaporation, and it reached the highest level in June. Then, soil salinity decreased precipitously due to leaching by rainwater in June, July and August, depending on the timing of the rainy season. Soil salinity increased again from spring to early summer due to salt accumulation on the soil surface. The shallow soil covered by snow had high salinity in winter. Salinity at 0 mol L^-1^ NaCl (no addition of NaCl) was low throughout the experimental period. Compared with low soil salinity, soils with high salinity retained more water, especially those with 1.5 mol L^-1^ NaCl. The seasonal fluctuation of soil temperature, moisture and salinity at depths of 2 and 5 cm was less intense than that at 0 cm. The trends of soil moisture and salinity differed between the 2 years due to differences in timing of precipitation in 2017 and 2018.

### Germination and depletion of the three type of diaspores

**Fig. 4:**
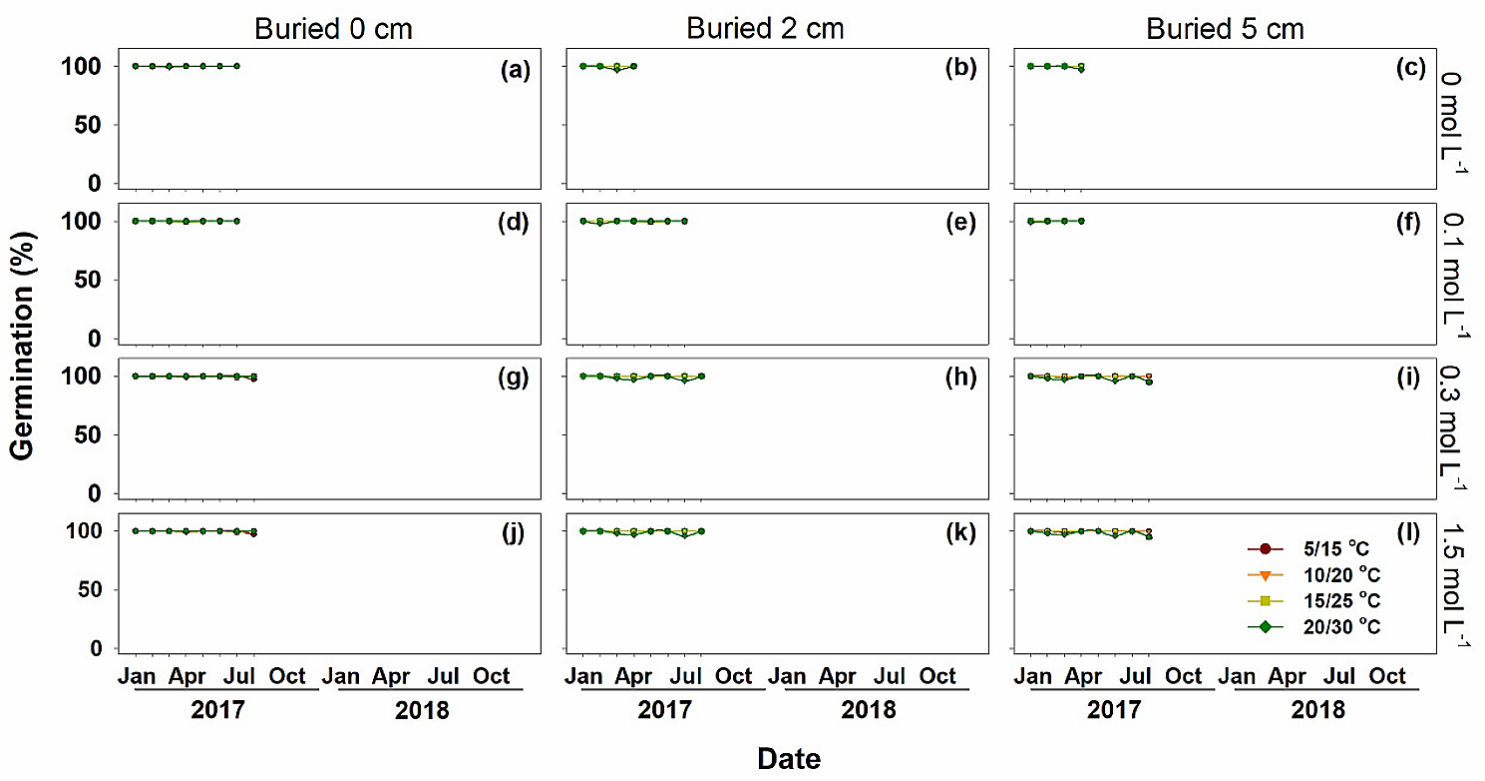
Mean monthly germination percentages (Mean ± SE) of *Atriplex centralasiatica* type A diaspores at four soil salt concentrations and three burial depths in the 2-year soil seedbank experiment. Four temperatures were used. The differents among each temperature was very small (almost all germinated to 100%). Thus, the line looks like only one temperature was used

**Fig. 5:**
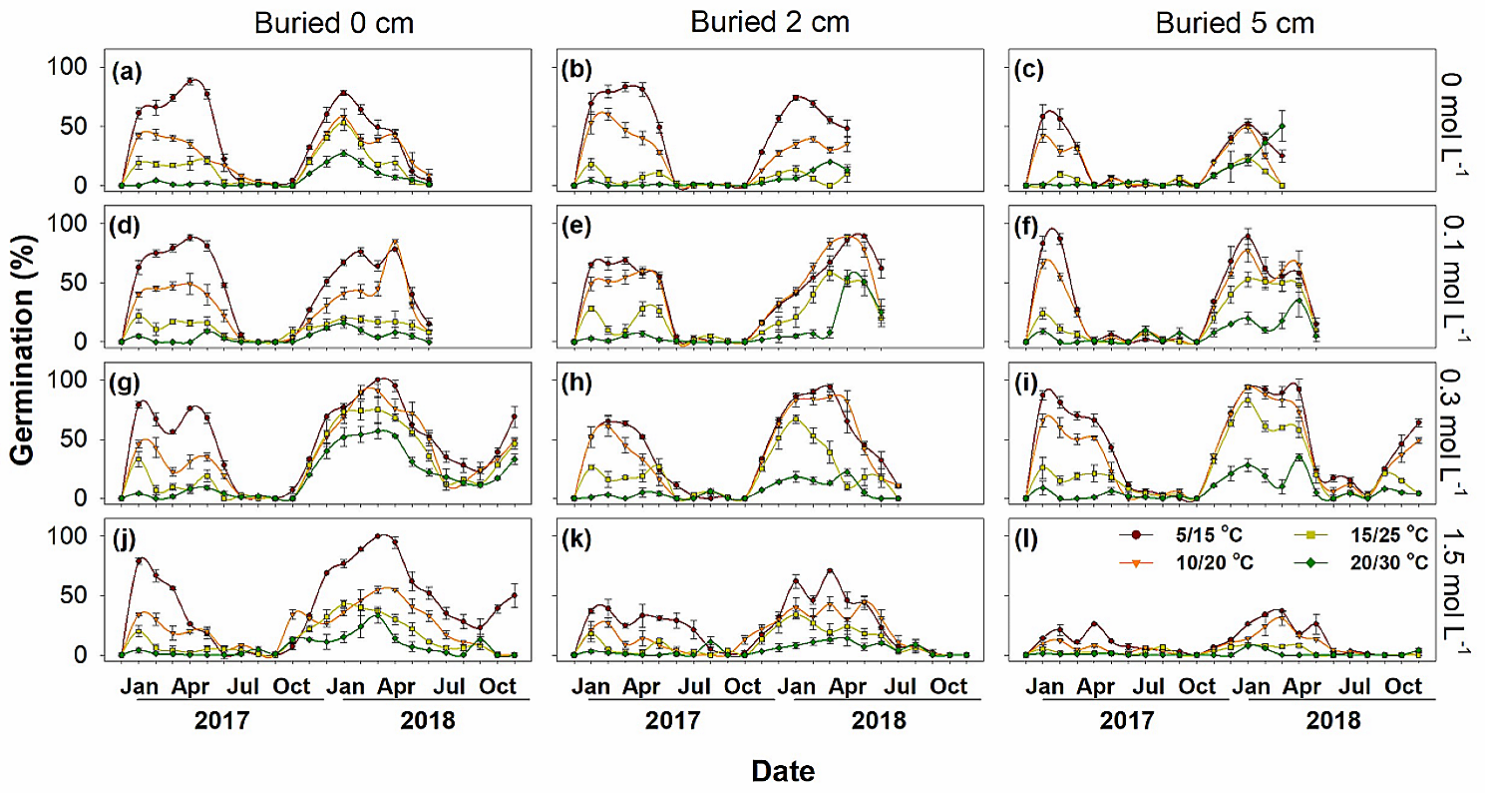
Mean monthly germination percentages (Mean ± SE) of *Atriplex centralasiatica* type B diaspores at four soil salt concentrations and three burial depths in the 2-year soil seedbank experiment

**Fig. 6:**
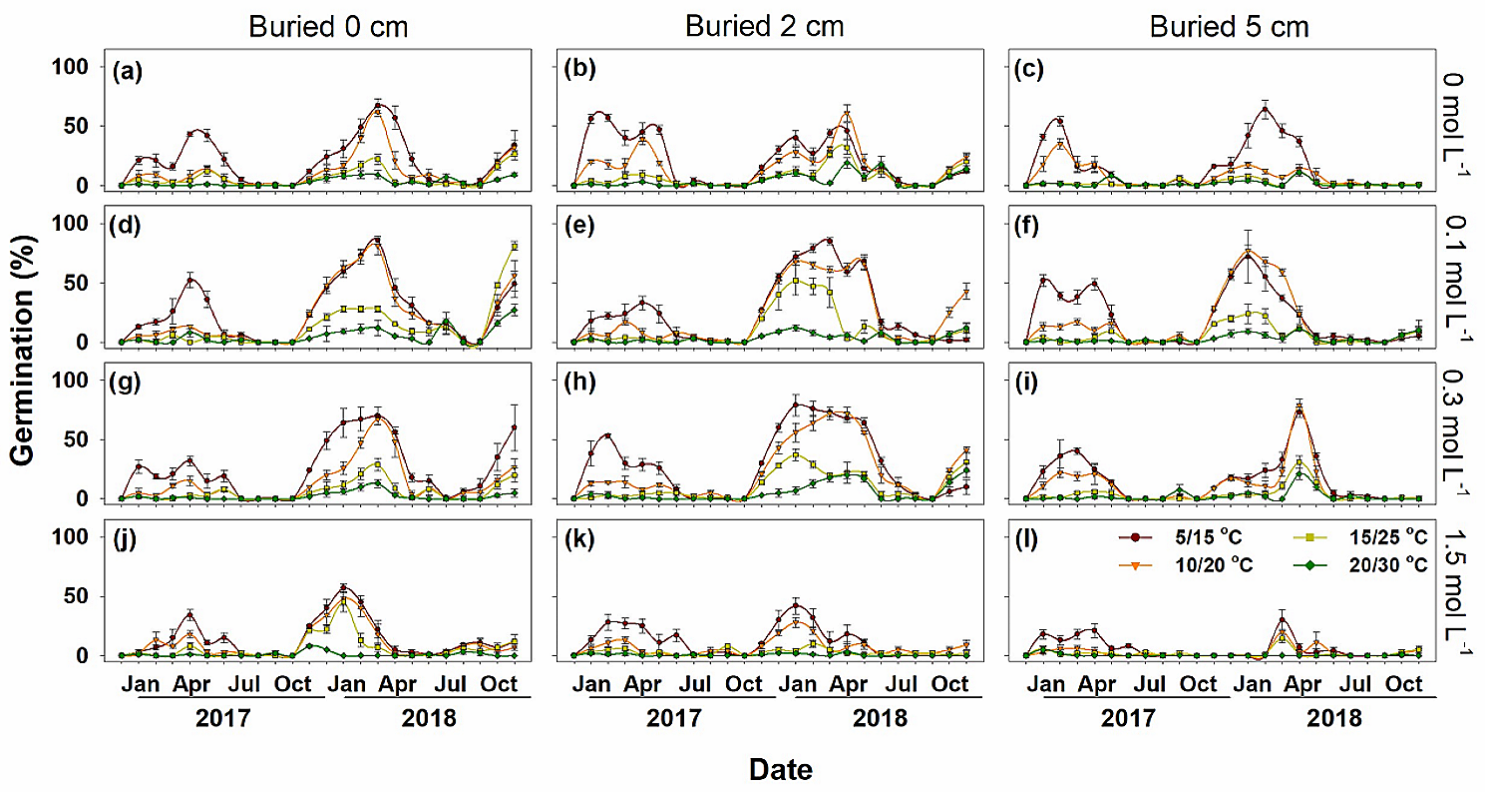
Mean monthly germination percentages (Mean ± SE) of *Atriplex centralasiatica* type C diaspores at four soil salt concentrations and three burial depths in the 2-year soil seedbank experiment

Germination differed significantly among the three types of diaspores. Type A diaspores retained high germination (96 ~ 100%) throughout the post-dispersal period, and all of them had germinated before October 2017 (Fig. 4). Type B germinated from 0% in the dormant season (freshly collected in September and in July and August in the following 2 years) to 100% in non-dormant season (from November to the following Feburary each year) (Fig. 5). Most type B diaspores had germinated before July 2018, except those buried in a high salt stress condition (1.5 mol L^-1^). Type C diaspores had much lower germination percentages during the dormancy cycle, and most of them remained in the soil seedbank during the 2-year burial experiment (Fig. 6).

### Germination of diaspores exhumed at different times of year

**Fig. 7:**
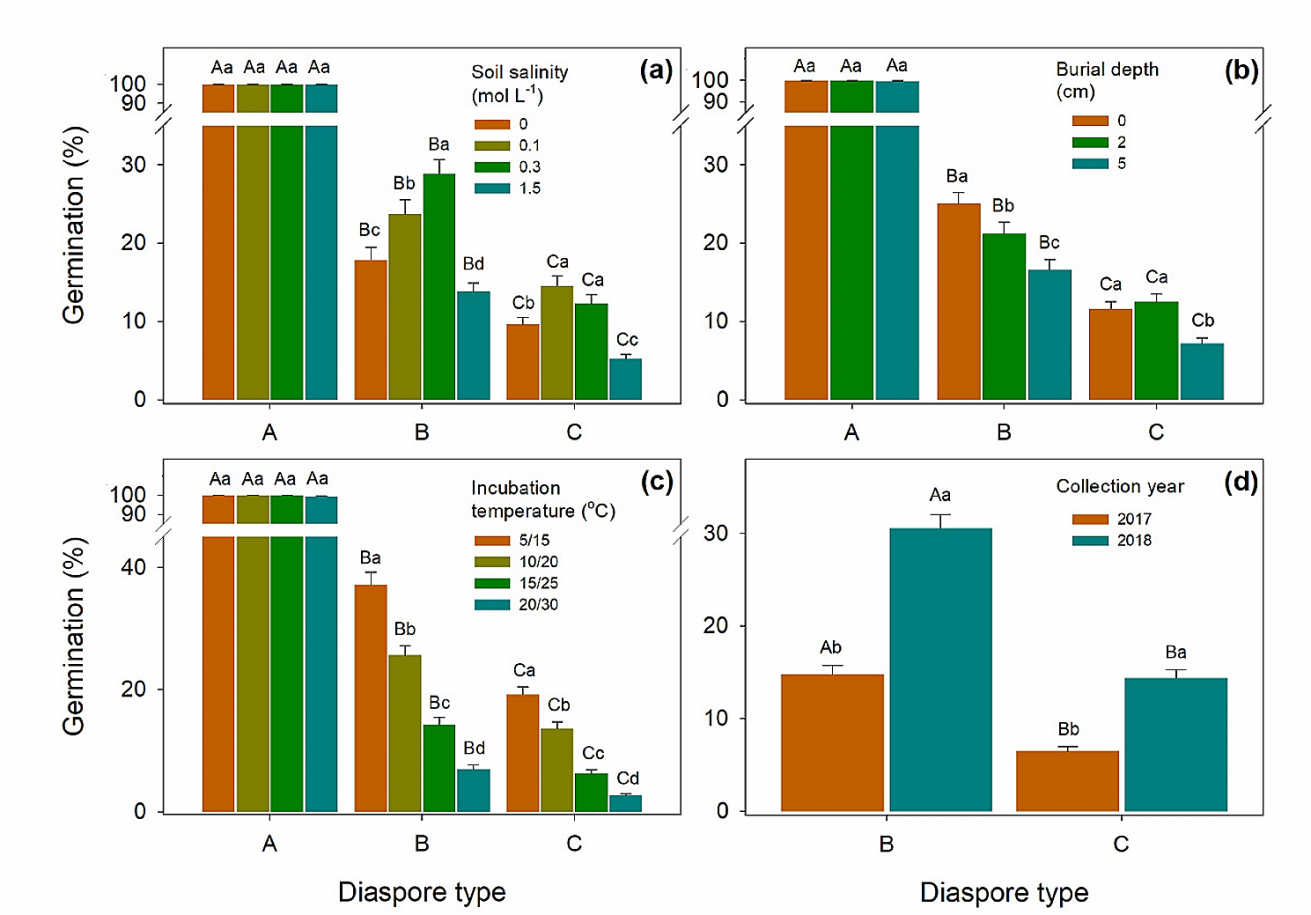
Effects of soil salinity, buried depth, incubation temperature and year of collection on germination of diaspore types A, B and C of *Atriplex centialasiatica* in the 2-year soil seedbank experiment. (**a**) Mean monthly germination percentages of type A, type B and type C at different concentrations of soil salinity. (**b**) Mean monthly germination of type A, type B and type C at different burial depths. (**c**) Mean monthly germination percentages of type A, type B and type C at different incubation temperatures. (**d**) Mean monthly germination percentages of type B and type C diaspores collected in 2017 and 2018. Different uppercase letters indicate significant differences in germination percentage among different diaspore types at the same salinity (**a**), burial depth (**b**), incubation temperature (**c**) and collection year (**d**) and different lowercase letters significant differences in germination among different salinity (**a**), burial depth (**b**), incubation temperature (**c**) and collection year (**d**) for the same diaspore type (*P* < 0.05)

Diaspores exhumed in January and February germinated to significantly higher percentages than those exhumed in other months (*P* < 0.01). Obvious dormancy cycles were exhibited by dormant types B and C diaspores, for which germination increased from September to February and then decreased from March to August (Figs. 5 and 6). Germination of the heteromorphic diaspores in 2018 was significantly higher than in 2017 in all treatments (*P* < 0.01) (Fig. 7d).

### Effects of soil salinity on germination

For type A diaspores exhumed from the pots, germination remained high in all soil salinity concentrations until all of them were depleted. Type B diaspores germinated similarly at 0, 0.1 and 0.3 mol L^-1^ soil salinity and decreased abruptly when soil salinity increased to 1.5 mol L^-1^, especially at the 2 cm and 5 cm burial depths (Fig. 7a). High salinity also narrowed the length of germination windows. Type C diaspores had a similar germination response as those of type B; however, type C was more sensitive to soil salinities than type B. The process of dormancy induction of Type C was much faster than that for type B, and 0.3 mol L^-1^ NaCl limited germination of type C. Once salt accumulation began in March, the germination window closed quickly. Peaks in germination percentages also decreased precipitously with the increase of buried depth and soil salinity. Salt concentrations of 0.1, 0.3 and 0.1 mol L^-1^ were the most suitable conditions for germination of type A, B and C, respectively (Fig. 7a). Germination of all diaspores types was significantly inhibited when buried in soil at 1.5 mol L^-1^ NaCl ( *P* < 0.01).

### Burial depth

Germination percentage decreased significantly with buried depth (*P* < 0.01). Diaspores on the soil surface germinated to much higher percentages than those buried at 2 cm and 5 cm (Fig. 7b). Type A germinated at all burial depths, but type B germinated only at burial depths of 2 cm and 5 cm in the first germination season. Germination percentages of type A and type B exhumed from the 5 cm depth was lower than those on the surface or exhumed from a soil depth of 2 cm (Figs. 4 and 5), while both 2 cm and 5 cm depths significantly inhibited germination of type C (*P* < 0.01) (Fig. 6).

### Incubation temperature regimes

For all three types of diaspores, germination decreased significantly (*P* < 0.01) with increased incubation temperature (Figs. 4, 5, 6 and 7c), and temperature inhibition of germination increased with level of dormancy depth of the three kinds of diaspores (i.e. type C > type B > type A). Only a few type B and type C diaspores germinated at 20/30°C, while > 80% of type A germinated at this temperature regime (Fig. 7c). However, type B and type C diaspores germinated to significantly higher percentages at 10/20°C and 15/25°C in the germination season (March to June) in 2018 than in 2017 in the field (*P* < 0.01).

### Dynamics of the number of viable diaspores in each pot

**Fig. 8:**
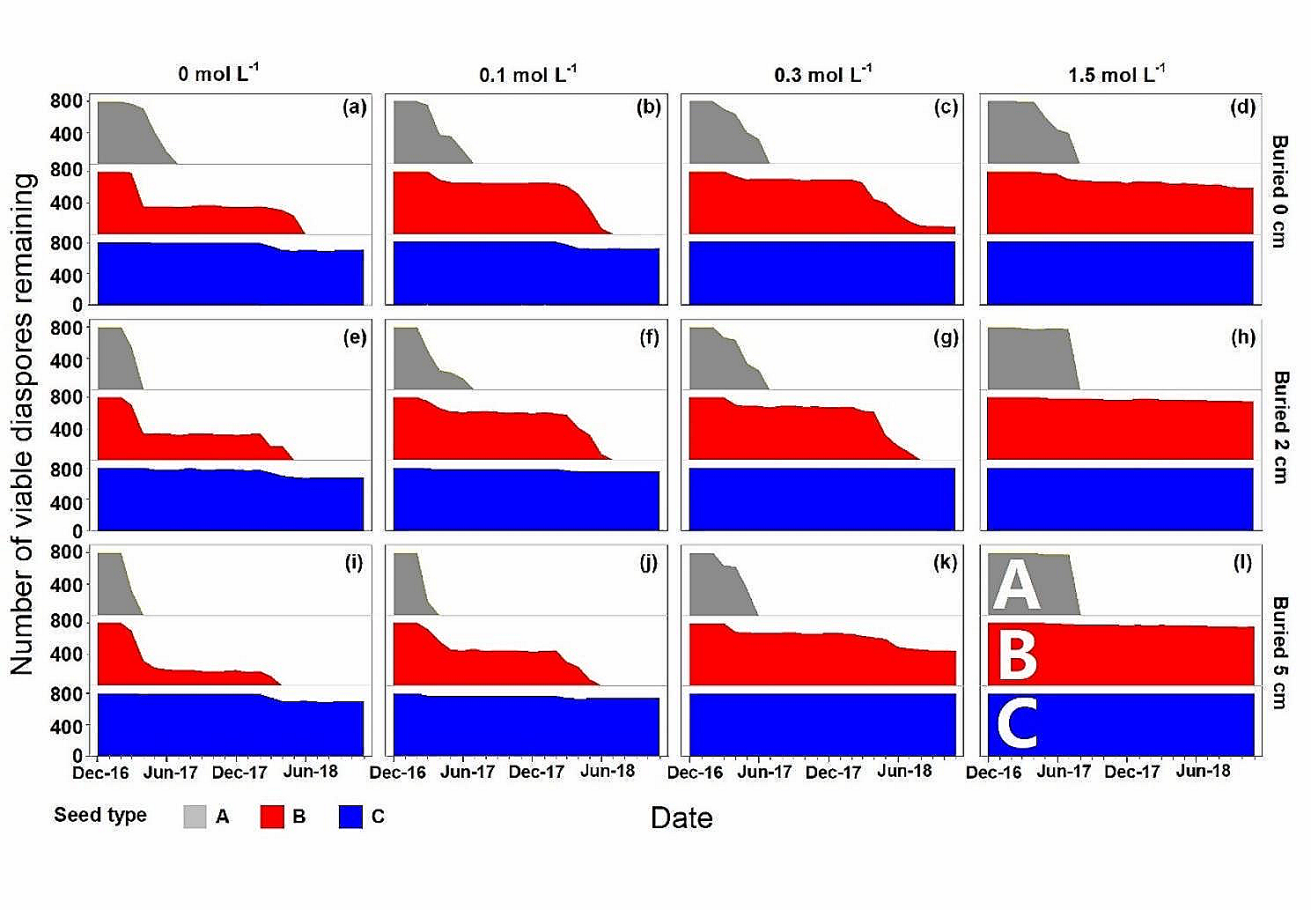
Mean number of types **A**, **B** and **C** diaspores of *Atriplex centralasiatica* remaining after various periods of burial at soil dpeths of 0 cm, 2 cm and 5 cm. Diaspores were buried in December 2016 and their presence/absence monitored until November 2018

Number of viable diaspores in each bag was counted each month. Survival percentages of type A and type B buried in the 0 and 0.1 mol L^-1^ NaCl addition treatments decreased with burial depth (Fig. 8a-i), and no viable type A diaspores remained in the 0–0.1 mol L^-1^ NaCl addition treatments in July 2017. The number of viable type A diaspores decreased sharply in July 2017, even in the 1.5 mol L^-1^ (Fig. 8d, h,l). All non-germinated type A diaspores buried in soil with 1.5 mol L^-1^ salinity were nonviable in October 2017. At a soil salinity ≤ 0.3 mol L^-1^, the number of viable diaspores decreased sharply in the second spring, except at 5 cm burial depth in 0.3 mol L^-1^. Only a few type C diaspores germinated or lost viability in the 2 years; the highest depletion (84.38%) occurred in 0.1 mol L^-1^ NaCl at the 2 cm burial depth.

## Discussion

Plant species have complex requirements for annually fluctuating environmental signals that ensure seedling recruitment [1, 28], and in our study soil temperature, soil moisture and soil salinity exhibited obvious annual fluctuations (Fig. S1). Germination and seed depletion of the three types of diaspores of *A. centralasiatica* also exhibited seasonal dynamics. Diaspore dormancy/germination and persistence/depletion of *A. centralasiatica* diaspores in the soil seedbank were influenced by the seasonal fluctuation of climatic conditions, which differed among the three diaspore types, soil salinity and burial depth. Burial at 5 cm, high salinity and non-salinity inhibited germination of non-dormant seeds of diaspore types B and C, while high-saline stress and shallow soil burial depth delayed depletion of diaspore A, B and C. Unlike most studies on halophytes in which optimum germination occurred in non-saline condition [29–35], diaspores of *A. centralasiatica* germinated better in low soil salinity than in non-saline soil.

Non-dormant type A diaspores retained high germination potential until they were depleted in the year following their maturity (Figs. 3 and 4), which can be definited as very fast germination seed types [11, 12]. Although high salt stress delayed germination of type A diaspores and increased their mortality, all of the remaining viable type A diaspores germinated to a high percentage at mid-growing season, when soil salinity concentration had been diluted to a very low level by rainwater and snow melt. The annual decrease in soil salt is a low salinity ‘window’ for the germination of type A diaspores. However, there is little chance for seedlings to complete their life cycle due to the short period of time of high temperatures and rainfall for seedling growth prior to onset of frost [10]. Although type A diaspores experienced seasonal soil salinlity stress, they are non-dormant and cannot be induced into secondary dormancy that would prevent their germination at the mid growing season [11, 25].

Dormancy cycle is commen in species with PD seeds, which delays germination and increases the local adaptation in harsh environments [7]. Unlike type A diaspores, potential germinability of types B and C cycled in response to seasonal environmental conditions, and it increased from September to the following March and decreased from April to August (Figs. 6 and 7). All type B diaspores were depleted, while only a few type C were depleted during 2 years of burial (Fig. 8). Temperature is the dominant seasonal signal that controls seed dormancy for most species inhabiting temperate environments [7, 36–38]. For dormant diaspores of *A. centralasiatica*, germination was restricted by high temperatures in the field, even in the moist non-saline conditions. To ensure completion of the life cycle, seedlings of *A. centralasiatica* should become established early in the growing season, when temperatures are low, since the warm rainy period is not long enough for plants to reproduce in the desert [11, 25]. This mechanism of controlling the timing of germination ensures the efficient utilization of seeds [7, 10, 14, 39]. Type C diaspores were more sensitive to temperature than type B (Figs. 6 and 7). Thus, less time and lower temperatures were needed to inhibit germination (i.e. secondary dormancy induced) for type C than type B diaspores. This phenotype indicates that the type C diaspores is at lower risk than the type B in seedling recruitments, since shadow ranges of environmental factors are needed in dormancy release.

For most species with heteromorphic seeds in Armaranthaceae, the (fresh) seeds of non-dormant morph have higher tolerance to salinity than small black ones [10, 39]. Production of non-dormant seeds is a “high-risk” strategy in arid and semi-arid habitatst, which could increase species competitiveness as well as and maximum colonization [15]. Although 1 month (December 2017) of cold stratification broke their dormancy, soil salinity modified the dormancy release process of diaspore types B and C in the field. Type A retained high germinability until they were completely depleted in less than 1 year, even in the highest salt stress conditions. However, even though incubated in distilled water, germination of types B and C diaspores collected in high saline soil was inhibited, since they were induced into secondary dormancy probably due to the lower water potentials [11]. After going through two dormancy cycles, the germination temperature window of type B diaspores had widened, and they germinated in the field in summer, like type A diaspores. Additionally, the best condition to break nondeep PD in seeds of halophytes is non-saline conditions generally [7, 30]. In our field experiment, the highest germination was in 0.3 and 0.1 mol/ L NaCl for type B and type C, respectively (Fig. 7). Type C diaspores have thick globular bracteoles [11]. After 2 years, the bracteoles of type B diaspores had diasppeared, leaving only the naked shiny seed. Widening of the window for germination may be a trade-off between the risk of seed aging and that of seedlings dying before they can complete their life cycle [15].

The time of dormancy release overlaps with the time of salt accumulation on the soil surface, from autumn to the following spring. Type C with deeper dormancy than that of type B also was much sensitive to salinity than type B, and less than 10% of seeds were depleted in 2 years. The long persistence of type C may be due to the local environment since salinity in dry and cold environments could prolong seed survival of halophytes [40]. Thus although high salinity increased death of diaspore type A, high salinity delayed diaspore depletion of *A. centralasiatica*, since a large portion of of total diapsores of this species are types B and C. Comparing with results from previous laboratory studies in which salinity induced seeds into secondary dormancy [26, 32], it seems that there is no evidence that soil salinity induces seeds of A. centralasiatica into secondary dormancy in the field. The results confirm our hypothesis that the seasonal dynamics of soil salinity play a crucial role in regulating seed dormancy/ germination and long-term viability of the soil seed bank of A. centralasiatica, thereby influencing these life-history traits.

Soil moisture, temperature and salinity, which correlate to seed germination, relative to soil depth [1, 2, 12]. For *A. centralasiatica*, the combined effect of soil moisture and salinity, both of which were affected by burial depth, may be the main factors regulating responses of seeds in the soil seedbank. Diaspores buried at 2–5 cm soil depths increased seed depletion via seedling emergence, although only a low proportion of types B and C diaspores were released from dormancy. Emergence from moist soil at 2 and 5 cm help ensure successful establishment of seedlings in the dry spring. As observed during our germinating tests, germination of types B and C took at least 2 and 5 days, respectively. Diaspore germination occurs only when precipitation is high enough for seedlings to become established. Diaspores buried at 5 cm would be in soil that was more moist than those at shallower burial depths, and the bracteoles on the diaspores could help retain moisture and thus play a role in inducing the enclosed seeds into dormancy. For *A. sagittata* [41] and *A. grithii* [42], solutions leached from bracteoles inhibited germination of naked seeds and induce them into secondary dormancy.

In conclusion, seasonal fluctuation of soil temperature and moisture controls seed dormancy/germination and persistence/depletion of soil seedbanks of *A. centralasiatica.* However, the responses of diaspores are modified by soil salinity and burial depth. This is the first report of adaptive mechanisms to different salinity concentrations and seasonal fluctuations of soil salinity for a diaspore heteromorphic halophyte. The heteromorphic diaspores differed in persistence and germinability in response to soil salinity and burial depth, which may be a bet-hadging strategy that allows *A. centralasiatic* to persist in an unpredictable seasonally dry environment. However, the study confined to artificially manipulated soil salt levels at a fixed site, necessitating future exploration into the mechanisms of how naturally occurring soil salt gradients across geographic scales influence soil seed banks in actual habitats.

### Electronic supplementary material

Below is the link to the electronic supplementary material.

Supplementary Material 1

## Data Availability

The data supporting the conclusions of this article is included within the article (and its additional file). All data generated or analyzed during this study are included in this article, and voucher specimens and diaspores of A. centralasiatica are available from the corresponding author upon request.
