# Supplementary material for: Soil salinity regulates spatial-temporal heterogeneity of seed germination and seedbank persistence of an annual diaspore-trimorphic halophyte in northern China
Source: BMC Plant Biol. 2024 Jun 26;24:604. doi: 10.1186/s12870-024-05307-x (PMC11201874; doi:10.1186/s12870-024-05307-x)

**Supplementary Figures**

**Fig. S1** Mean soil temperature, soil moisture and soil salinity at 0, 2 and 5 cm in each of 24 pots from December 2016 to November 2018. Each row represents the depth at which sensor was buried and each column the salinity in the pot in which the sensor was buried. Sensors were buried at 0.5 cm to measure the soil condition at the soil surface (0 cm), since soil moisture and soil salinity cannot be detected if sensors were placed on the soil surface.

**Fig. S1**


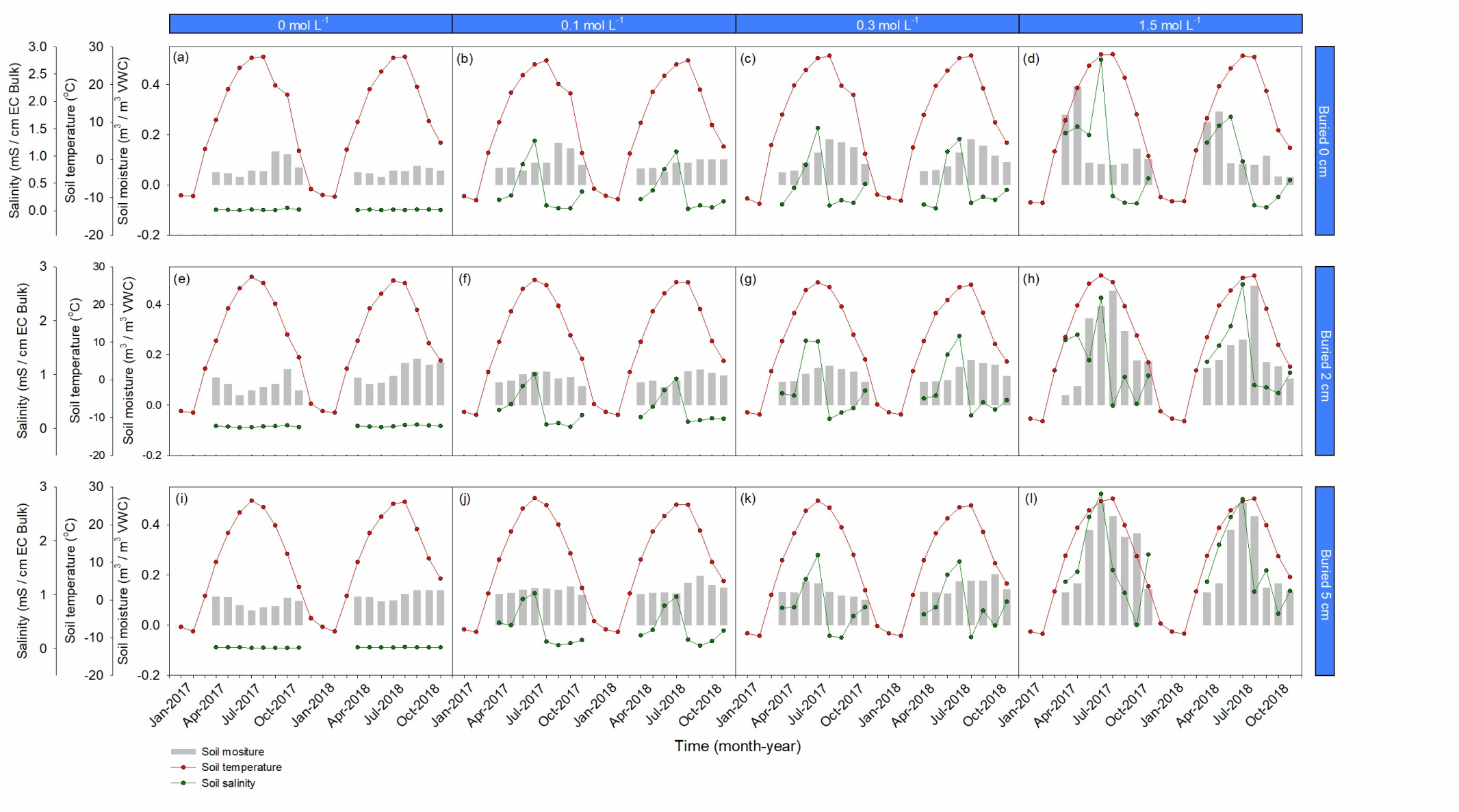

Supplement: Supplementary file 1 — Supplementary Material 1 [file 12870_2024_5307_MOESM1_ESM.docx]
